# A Novel Method for Analysing Frequent Observations from Questionnaires in Order to Model Patient-Reported Outcomes: Application to EXACT® Daily Diary Data from COPD Patients

**DOI:** 10.1208/s12248-019-0319-9

**Published:** 2019-04-26

**Authors:** Eva Germovsek, Claire Ambery, Shuying Yang, Misba Beerahee, Mats O. Karlsson, Elodie L. Plan

**Affiliations:** 1 Department of Pharmaceutical Biosciences, Uppsala University, Box 591, SE-751 24 Uppsala, Sweden; 2 Clinical Pharmacology Modelling and Simulation, GlaxoSmithKline, London, UK

**Keywords:** EXACT questionnaire, IRT, Markov model, NONMEM, PRO

## Abstract

Chronic obstructive pulmonary disease (COPD) is a progressive lung disease with approximately 174 million cases worldwide. Electronic questionnaires are increasingly used for collecting patient-reported-outcome (PRO) data about disease symptoms. Our aim was to leverage PRO data, collected to record COPD disease symptoms, in a general modelling framework to enable interpretation of PRO observations in relation to disease progression and potential to predict exacerbations. The data were collected daily over a year, in a prospective, observational study. The e-questionnaire, the EXAcerbations of COPD Tool (EXACT®) included 14 items (*i.e.* questions) with 4 or 5 ordered categorical response options. An item response theory (IRT) model was used to relate the responses from each item to the underlying latent variable (which we refer to as disease severity), and on each item level, Markov models (MM) with 4 or 5 categories were applied to describe the dependence between consecutive observations. Minimal continuous time MMs were used and parameterised using ordinary differential equations. One hundred twenty-seven COPD patients were included (median age 67 years, 54% male, 39% current smokers), providing approximately 40,000 observations per EXACT® item. The final model suggested that, with time, patients more often reported the same scores as the previous day, *i.e.* the scores were more stable. The modelled COPD disease severity change over time varied markedly between subjects, but was small in the typical individual. This is the first IRT model with Markovian properties; our analysis proved them necessary for predicting symptom-defined exacerbations.

**Electronic supplementary material:**

The online version of this article (10.1208/s12248-019-0319-9) contains supplementary material, which is available to authorized users.

## INTRODUCTION

Chronic obstructive pulmonary disease (COPD) is an inflammatory disease of the lung, characterised by airflow obstruction that progresses with time. The most important risk factor associated with COPD is considered smoking, but risk factors also include other exposures (*e.g.* air pollution, occupational dusts and chemicals) and host factors, such as α1-antitrypsin deficiency (1). COPD is associated with emphysema and mucus hypersecretion, and its progression is punctuated with acute periods of a temporary increase in symptoms, also called exacerbations (2,3).Historically, exacerbations are defined by a clinic visit or hospitalisation with medical treatment (clinically confirmed); however, recently, questionnaires have been validated as useful for symptom-defined exacerbations (4–6). Exacerbations contribute to an accelerated decline of pulmonary function, higher risk of cardiovascular events (7) and worse quality of life (8) and are a major cause of COPD-related hospital admissions, morbidity and mortality; therefore, also increasing healthcare costs. Approximately 174 million people had COPD in 2015 (9), and around three million die from it every year (10). The disease burden of COPD is third worldwide (11,12), but may even increase in the future, due to an ageing society.

For slowly progressing diseases, such as COPD, clinical trials that investigate symptom-based exacerbations and/or disease progression are often relatively long (*e.g.* a year, or more), making collection of multiple observations possible. Modelling techniques facilitate using all the information from the repeated measurements and can therefore provide additional insight over and above the output from traditional statistical methods, where typically only end-of-treatment information is used. Such longitudinal analysis has the advantage of quantifying any trend in response and may therefore be helpful in describing and predicting future exacerbations, which may be especially useful in early clinical trials. Most models published so far are either Markov models, focusing on the cost-effectiveness of the COPD treatment intervention (13–17), or logistic regression models, predicting the probability of exacerbation within the next 24 months (18) or COPD-patient hospital admissions (19). Some linear and non-linear regression models were also developed, aiming to predict disease progression (20–22). To our knowledge, none of the models published so far described the longitudinal COPD progression using daily patient-reported-outcome (PRO) data, which might be able to reflect symptoms earlier than, for example, clinicians’ reports (23). A model that would use daily PRO data in its entirety to predict changes in patient disease severity would therefore be valuable in assessing disease progression.

PRO data generally reflect health status reports that come directly from the patient and are being increasingly used to inform clinical decisions and assess improvements in a patient’s health status, and also in drug development (23–25). The standard method for analysing longitudinal PRO data from questionnaires (26) is the total instrument score, calculated from the individual item scores; hence, a single continuous variable is analysed. An alternative approach is to use PRO data in its entirety by developing a longitudinal mixed-effects item response theory (IRT) model (26), where the contribution of each individual item score is modelled separately over time, but related to a common underlying hypothetical latent variable (*i.e.* in IRT terminology and hereafter referred to as ‘disease severity’), which varies between individuals and over time (27). This can help one understand change in disease severity over time (*i.e.* symptom-based disease progression), particularly in early clinical development. Furthermore, as each individual item score is modelled separately giving rise to a unique ‘item characteristic curve’ (ICC), IRT modelling can provide knowledge on the item that is most informative for a population with a certain disease severity, which can be valuable information for clinicians applying the questionnaire.

Frequent observations of categorical data make the presence of the Markovian elements, typically identified as many consecutive same-item-score observations, likely. With daily data collection in studies, through the use of electronic devices, such correlations are likely to be manifested. In previously published implementations (28–35), IRT models assume independence under the structural model. This means that the probability distribution of outcomes are driven by the latent variable, and the ICCs only and observations, even when close to each other in time, are independent realisations. This is found often to not be the case with subjective scoring, and models to account for the dependence between consecutive observations in the data would be valuable.

In the present work, we analysed the PRO data, collected in the Acute Exacerbation and Respiratory InfectionS in COPD (AERIS) study (36,37), using the EXAcerbations of COPD Tool (EXACT®) (38,39), with two aims: (i) to create a model that can be useful as a basis for assessing disease progression including predictions of symptom-defined exacerbations and effect of treatment on these clinical trials, and (ii) to extend the IRT modelling methodology to incorporate the situation where underlying data display Markovian features.

## METHODS

### Data

The EXACT® PRO data were collected in the AERIS study (36,37), a prospective 2-year longitudinal observational study where adult and elderly patients were only receiving standard-of-care treatment, no investigational drug. The study was conducted at the Southampton General Hospital, UK (clinicaltrials.gov number NCT01360398). Details of study conduct and inclusion and exclusion criteria are reported elsewhere (36,37,40). Only the first-year data were available and analysed in the present work. The baseline characteristics of the patients included in the analysis were the following: age, gender, educational and professional status, smoking status, severity of COPD according to the Global Initiative for Chronic Obstructive Lung Disease (GOLD) staging, number of years with COPD, forced vital capacity (FVC), forced expiratory volume in the first second (FEV1).

The EXACT® questionnaire, completed on an electronic diary, was used daily to record patients’ answers to the 14 questions (9 with 5 ordered categorical response options (hereafter referred to as ‘categories’) and 5 with 4 categories, see Table I), to collect data on symptoms suggestive of an exacerbation of COPD. A higher item score indicated a more severe symptom, for example 0 (not at all), 1 (slightly), 2 (moderately), 3 (severely) and 4 (extremely). Due to the design of the study (specifically the device including the electronic questionnaire), partial completion of the questionnaire was not possible, but a missing day where a subject gave no answer to any of the items was possible. No data were excluded from the analysis.

**Table I:** Individual items from the EXACT® questionnaire, with their short description, and item-score scale, ordered by their chronic obstructive pulmonary disease symptom domain

Item	Description	Scale	Domain
7	Breathless	0–4	Breathlessness
8	How breathless	0–3	Breathlessness
9	Breathless—personal care	0–4	Breathlessness
10	Breathless—indoor activities	0–3	Breathlessness
11	Breathless—outdoor activities	0–3	Breathlessness
1	Congested chest	0–4	Chest symptoms
5	Chest discomfort	0–4	Chest symptoms
6	Chest tightness	0–4	Chest symptoms
2	Cough frequency	0–4	Cough and sputum
3	Mucus quantity	0–3	Cough and sputum
4	Sputum difficulty	0–4	Difficulty with sputum
12	Tired or weak	0–4	Tired or weak
13	Disturbed sleep	0–4	Sleep disturbance
14	Scared or worried	0–3	Psychological state

*Full question for each item from the questionnaire is provided elsewhere* *(* 41 *)* *. A higher score denotes a more severe symptom*

### Model Building

An IRT framework was combined with the Markov component, and the joint model was fitted to all available data simultaneously. For ease of model explanation, we firstly focus on the IRT part and then on how it is connected to the Markov part of the model.

An IRT model was developed to relate the responses from each individual item (items were ordered categorical data with 4 or 5 categories (Table I)) to an unobserved latent variable (42), *i.e.* the underlying COPD disease severity. All longitudinal data were used when modelling and also when establishing the item characteristic curves.

A logistic transformation was used, where the (steady-state) probability P of a subject *i* reporting a response at or above category (or, item-score) *k* is expressed as follows (Eq. 1). The steady-state probability of an item response being exactly *k* was given as described in Eq. 2.

1 $$ {P}_{ss}\left({Y}_{ij}\ge k\right)=\frac{\exp \left({a}_j\bullet \left({D}_i-{b}_{j,k}\right)\right)}{1+\exp \left({a}_j\bullet \left({D}_i-{b}_{j,k}\right)\right)}, $$

2 $$ {P}_{ss}\left({Y}_{ij}=k\right)={P}_{ss}\left({Y}_{ij}\ge k\right)-{P}_{ss}\left({Y}_{ij}\ge k+1\right), $$

where *D*_*i*_ is the disease severity of subject *i*, and *a*_*j*_ and *b*_*j*_ are parameters specific to item *j*; more specifically, *a*_*j*_ is the slope (or discrimination parameter), and *b*_*j,k*_ is the difficulty parameter for the item-score *k*, which was constrained to be increasing for increasing scores of the same item, namely *b*_*j,k* + 1_ ≥ *b*_*j,k*_. The parameter describing the COPD severity (*D*_*i*_) was not bounded and was assumed to follow a normal distribution *N*(0,1) at baseline (*D*_*i,t =* 0_).

The longitudinal changes in the COPD progression were modelled using a linear change in disease severity, as shown below in Eq. 3.

3 $$ {D}_i={D}_{i,t=0}+{\mathrm{slope}}_i\times t, $$

where slope_*i*_ is a subject-specific parameter with interindividual variability assumed to follow a normal distribution, and *t* is time in days.

To allow for the lack of independence between neighbouring observations, the Markov models were used on an individual item level. First-order MM was assumed, *i.e.* the next observation depended only on the current observation. The distribution of probabilities across the 4/5 categories were described using a set of 4/5 corresponding states. Changes in probabilities with time were described using a set of 4/5 ordinary differential equations (ODEs) where first-order transfer constants governed the changes in probability between adjacent states. As previously described (43), a reparameterisation allowed the Markovian component across all state transitions to be described in a single parameter, the mean equilibrium time (MET), which is the time when the dependency between observations does not change anymore. As the Markovian features are expressed through one parameter only, and because time is treated dynamically, this type of model has been referred to as a ‘minimal Continuous Time Markov Model (mCTMM)’ (43). The probability of the first observed score of an item was estimated as *P*_ss_ (*Y*_*ij*_ = *k*) at steady state given by Eqs. 1 and 2 above, *i.e.* without an assumption on the previous score. For all subsequent observations, the probabilities were reset immediately after each observation to represent the known distribution of probabilities, *i.e.* 1 for the currently observed state and 0 for all other states. The relation between MET and the rate constants (*λ*) and the steady-state probabilities (*P*_*ss*_) is given in Eqs. 4–6 below. Since transition times and rate constants are in inverse relationship, this means that as MET increases, the *λ* decreases, and therefore, the probability of transitions also decreases. In other words, this means that with an increase in the MET parameter, the Markovian properties of the system are becoming more important.

4 $$ MET={\left({\lambda}_{\left(k-1,k\right)}+{\lambda}_{\left(k,k-1\right)}\right)}^{-1}={\left({\lambda}_{\left(k,k+1\right)}+{\lambda}_{\left(k+1,k\right)}\right)}^{-1}, $$

5 $$ {\lambda}_{\left(k,k+1\right)}={\left( MET\times \left(1+\frac{P_{ss}\left({Y}_{ij}=k\right)}{P_{ss}\left({Y}_{ij}=k+1\right)}\right)\right)}^{-1}, $$

6 $$ {\lambda}_{\left(k+1,k\right)}={\lambda}_{\left(k,k+1\right)}\times \frac{P_{ss}\left({Y}_{ij}=k\right)}{P_{ss}\left({Y}_{ij}=k+1\right)}. $$

The ODEs used to describe the model are provided in Eqs. 7–11, with simplified notation for probabilities.

7 $$ \frac{d{P}_{k=0}}{dt}=-{P}_{k=0}\times {\lambda}_{\left(k=0,k=1\right)}+{P}_{k=1}\times {\lambda}_{\left(k=1,k=0\right)}, $$

8 $$ \frac{d{P}_{k=1}}{dt}=-{P}_{k=1}\times \left({\lambda}_{\left(k=1,k=0\right)}+{\lambda}_{\left(k=1,k=2\right)}\right)+{P}_{k=0}\times {\lambda}_{\left(k=0,k=1\right)}+{P}_{k=2}\times {\lambda}_{\left(k=2,k=1\right),} $$

9 $$ \frac{d{P}_{k=2}}{dt}=-{P}_{k=2}\times \left({\lambda}_{\left(k=2,k=1\right)}+{\lambda}_{\left(k=2,k=3\right)}\right)+{P}_{k=1}\times {\lambda}_{\left(k=1,k=2\right)}+{P}_{k=3}\times {\lambda}_{\left(k=3,k=2\right),} $$

10 $$ \frac{d{P}_{k=3}}{dt}=-{P}_{k=3}\times \left({\lambda}_{\left(k=3,k=2\right)}+{\lambda}_{\left(k=3,k=4\right)}\right)+{P}_{k=2}\times {\lambda}_{\left(k=2,k=3\right)}+{P}_{k=4}\times {\lambda}_{\left(k=4,k=3\right),} $$

11 $$ \frac{d{P}_{k=4}}{dt}=-{P}_{k=4}\times {\lambda}_{\left(k=4,k=3\right)}+{P}_{k=3}\times {\lambda}_{\left(k=3,k=4\right)}, $$

where, in the case of items with only 4 possible scores, rate constants *λ*_(*k* = 3,*k* = 4)_ and *λ*_(*k* = 4,*k* = 3)_ were assumed to be 0.

**Fig. 1:**
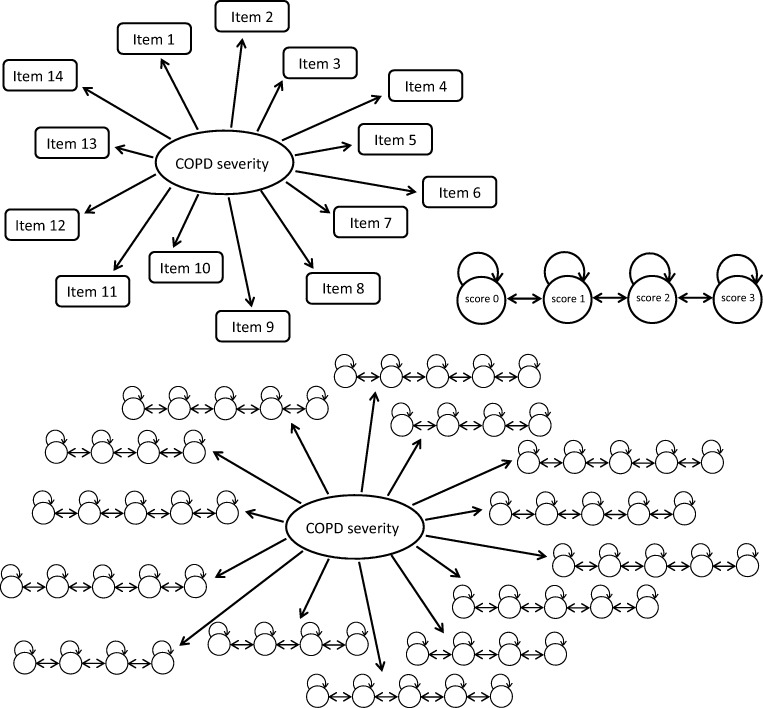
Schematic representation of the item response theory model (IRT) (upper left), Markov model (MM) for an item with 4 possible scores (upper right), and the whole model (*i.e.* IRT with a MM for each item) (below).

In the model, it was assumed that there was interindividual variability in MET expressed through an exponential distribution. A schematic representation of the IRT and Markov model(s) is shown in Fig. 1.

To obtain informative initial estimates for the parameters in the 4/5-compartment ODE model, an analytical solution (AS) for a 3-compartment ODE system was used. The data from each item were merged into 3 categories in 3 different combinations ((a) 0, 1, 2–4; (b) 0–1, 2, 3–4; and (c) 0–2, 3, 4), then fitted with the 3-compartment AS models, and the final estimates used as initial values in the 4/5-compartment ODE model.

To confirm that the Markov elements were needed in the model, a model without the Markov elements was tested, by fixing MET to a small value (0.1 days). Furthermore, item-specific MET, time-dependent MET (in a linear and a power fashion), and a Box-Cox transformed MET interindividual variability were tried as extensions to the model.

### Software and Estimation Method

NONMEM version 7.3 (ICON Development Solutions, Ellicott City, MD) (44) was used for modelling and simulation, together with the Laplace approximation to obtain the likelihood. Parameters of the joint model were estimated in a simultaneous fit. R (The R Foundation for Statistical Computing) (45), PsN (46) and R packages, such as, dplyr (47), Xpose4 (48) and ggplot2 (49) were used for data management, summary statistics and graphical examination of NONMEM outputs.

### Model Discrimination and Evaluation

To discriminate between the models and evaluate the final model, goodness-of-fit and visual predictive checks (VPC) were used, and the uncertainty on the model parameters was inspected. The models were also compared according to the objective function value (OFV) provided by NONMEM, where the difference in OFV (ΔOFV) is *χ*^2^ distributed, *i.e.* a ΔOFV > 3.84 corresponds to *p* < 0.05, for a one degree of freedom difference between two models. For each VPC, 1000 datasets were simulated using parameter estimates from the model, and 95% confidence intervals around key percentiles were computed. VPCs were produced on the individual item-score level, stratified and non-stratified by individual items, and on the total score level (*versus* time on study, and *versus* age), also stratified by covariates, such as gender. The total score was obtained as specified in the EXACT® manual (41), *i.e.* a 0–100 logit transformation was used. Additionally, a VPC of transitions was made, taking into account both the current and the previous state in order to evaluate the Markov part of the model.

### Model Application

To quantify the correlation between items, item-specific residuals (*RES*_*ij*_) were calculated as specified in the following Eqs. 12 and 13:

12 $$ {IPRED}_{ij}={\sum}_k^0P\left({Y}_{ij}=k\right)\times k, $$

13 $$ {RES}_{ij}={DV}_{ij}-{IPRED}_{ij}, $$

where *DV*_*ij*_ is the response of subject *i* to item *j*, and *IPRED*_*ij*_ is the corresponding weighted prediction (32).

Furthermore, the Fisher information (*i.e.* the second derivative of the log-likelihood) (50) for each item was obtained, in order to examine which item provides the most information for the following: (a) a typical subject from this study, (b) the ‘healthier’ part of the population (*i.e.* 5th percentile) and (c) the ‘sicker’ part (*i.e.* 95th percentile) of the population.

### Simulations

To further evaluate the model, we simulated clinical outcomes used in COPD treatment, such as a symptom-defined exacerbation event. Due to complexity, a simplified version of the definition of a symptom-defined event from the EXACT® manual (41) was used. Specifically, a symptom-defined exacerbation event was defined as an increase in total score over baseline of at least 12 points over 2 days, or of at least 9 points over 3 days; if baseline was zero, there was no event. Baseline was reset every 4 weeks. The baseline for the first block of 4 weeks was determined as the mean total score in the first week of the study, and for subsequent 4-week-block baselines, the mean total score of the last week of the previous 4-week block was used. If a subject completed the questionnaire for fewer than 4 days in the ‘last week’, baseline was not reset. The cumulative proportion of subjects that already had a symptom-defined exacerbation event was computed for both observed and simulated data, and the agreement between model-predicted (from *n* = 100 simulations) and observed symptom-defined exacerbations was assessed.

## RESULTS

### Data

Data from 127 COPD patients (median age 67 years, 54% male, 39% current smokers at study initiation) were available and included approximately 40,000 observations per item. Baseline characteristics of the study participants are presented in Table II. Before the end of the first year, 36 subjects (28%) stopped filling in their questionnaire, 22 of which discontinued the study due to reasons not directly related to the disease severity (37). Five subjects had no missing days (*i.e.* days when they did not fill in the e-questionnaire), and the remaining 122 subjects had a median (range) 13 (1–221) total missing days. The median (range) consecutive missing days for all subjects was 3 (0–74) days.

**Table II:** Baseline characteristics of the subjects in the study

	Median (range) or count (%)
Total number of subjects (*n*)	127
Age (years)	67 (42–85)
Time with COPD (years)	7 (0–45)
FVC (L)	3.25 (1.61–4.99)
FEV1 (L)	2.54 (1.27–4.08)
Sex: male (*n*)	68 (54%)
Profession (*n*)	Retired: 99(78%), full-time employed: 9(7%), part-time employed: 8(6%), on sick leave: 6(5%), unemployed: 4(3%), self-employed: 1(1%)
Education (*n*)	GCSE: 32(25%), A-level: 2(2%), higher education: 26(20%), University: 7(6%), other: 60(47%)
Smoking status: smoker (*n*)	50 (39%)
COPD GOLD disease status (see reference (51)) (*n*)	Moderate: 57(45%), severe: 51(40%), very severe: 19(15%)

*COPD is chronic obstructive pulmonary disease, FVC is forced vital capacity, FEV1 is forced expiratory volume in 1 s, GCSE is General Certificate of Secondary Education. GOLD is the Global Initiative for Chronic Obstructive Lung Disease*

### Modelling

**Fig. 2:**
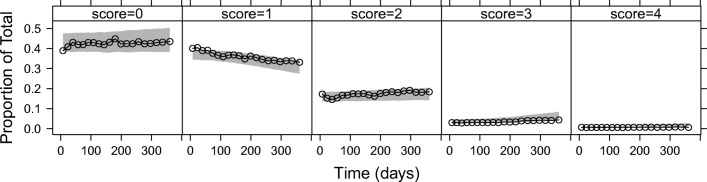
Visual predictive check for item scores of all 14 items, showing different proportions of observations (black lines) with the corresponding 95% confidence interval (grey area) from 1000 simulations.

**Fig. 3:**
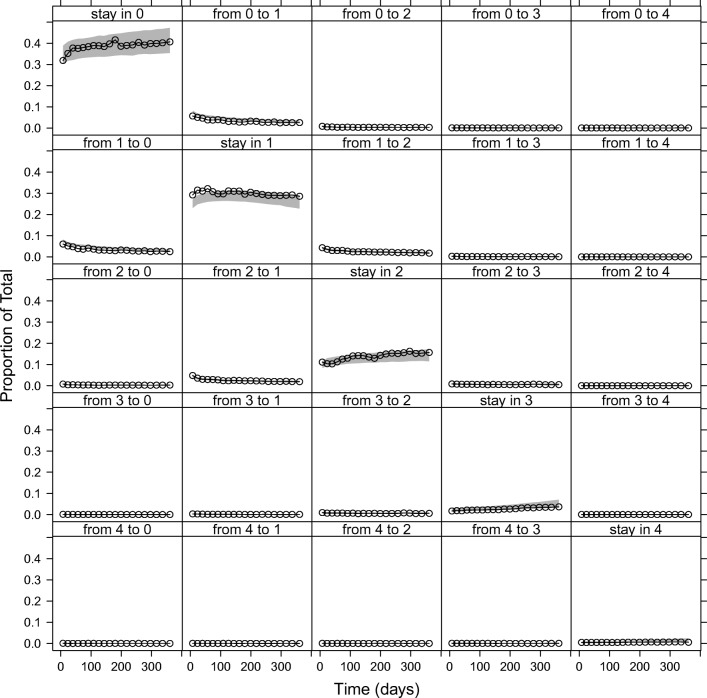
Visual predictive check for all 14 items, showing different proportions of observed transitions (black lines) with the corresponding 95% confidence intervals (grey areas) from 1000 simulations. Transitions are described in the panels.

An IRT model with 14 (4/5-compartment) the Markov sub-models (supplementary material) was successfully fitted to the data (Figs. 2, 3 and4). The model without the Markov elements performed much worse than the model with the Markov elements, *i.e.* it was not able to predict several transitions as seen on the transition VPC (Fig. S1, supplementary material).

**Fig. 4:**
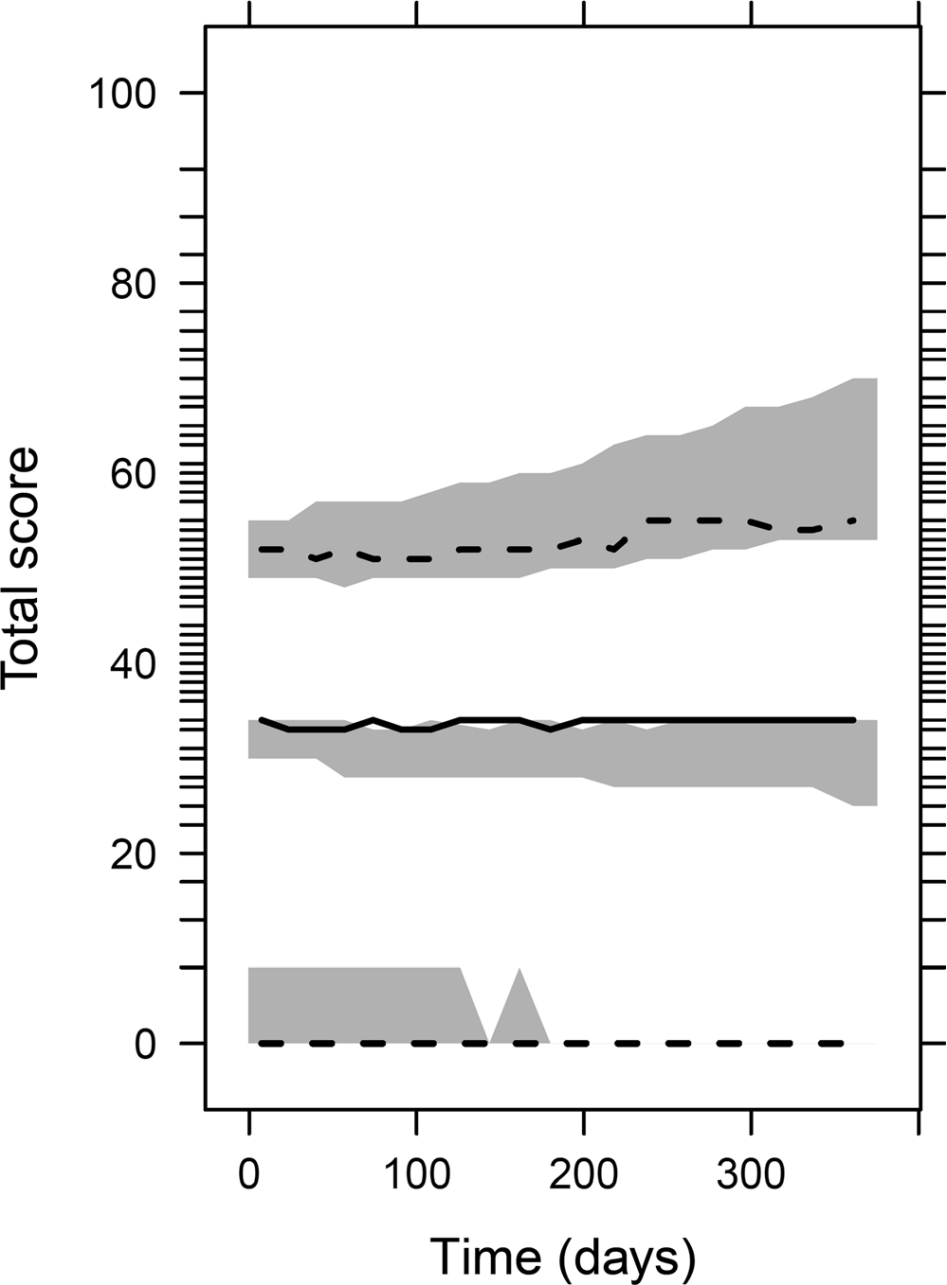
Visual predictive check (*n* = 1000) for the total score (transformed to a 0–100 scale), *i.e.* sum of all individual item scores. Black lines represent data (2.5th, 50th, 97.5th percentiles), and grey areas are the corresponding 95% confidence intervals from 1000 simulations. Lack of a grey-shaded area indicates that the 95% confidence interval of simulations fully agrees with the observed data.

The base model was an IRT model with the Markov elements, and a single non-changing MET parameter. Among the extensions to the model, linear time-dependency on MET improved the visual diagnostics the most and resulted in the biggest OFV drop (ΔOFV = 8727, compared to the base model) among the tested models. For example, when using the model with 14 item-specific METs, the visual diagnostics did not improve, and the OFV drop was lower (ΔOFV = 4678); similarly for the models with a Box-Cox transformation of the variability on MET (ΔOFV = 13.7), or a power time-dependency on the MET parameter (ΔOFV = 5902), all compared to the base model. Therefore, the model with a linear time-dependency on MET was chosen as the final model.

Final parameter estimates are presented with uncertainty in Table S2 (supplementary material). The mean (standard error) equilibrium time was estimated as 1.2 (0.07) days at the beginning of the study (*i.e.* day 0), and 5.1 (0.57) days at the end of the study (*i.e.* day 365). The mean (standard error) slope on disease severity representing disease progression was 0.007 (0.08) latent variable scale units per year, with substantial interindividual variability, 122 %CV (Table S2).

Visual predictive checks on the item score level showed satisfactory fit to the data (Fig. 2; stratified by an individual item, Figures S3a-d, supplementary material). A VPC of transitions is shown in Fig. 3, and a VPC of the total scores plotted against time in Fig. 4 (also stratified by gender, and *versus* age are shown in Figures S4, and S5, respectively, in the supplementary material).

### Model Application

**Fig. 5:**
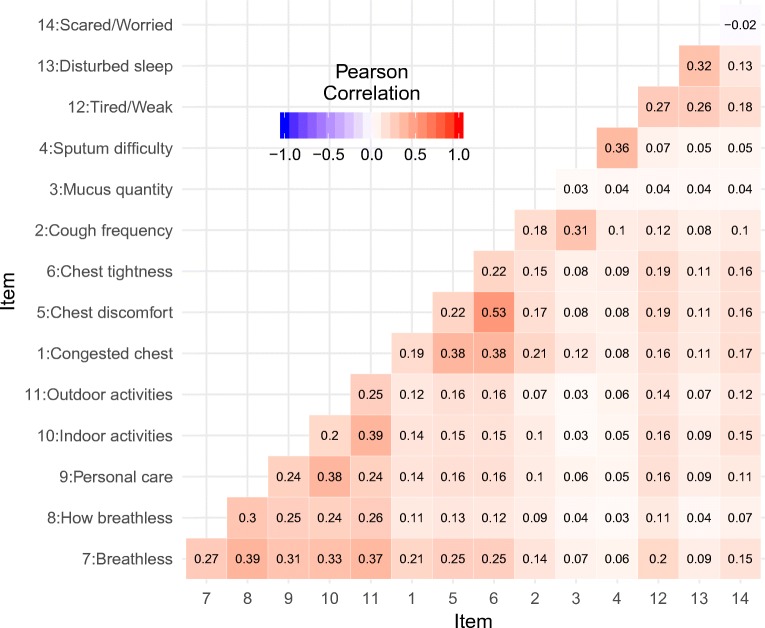
A heat map showing Pearson correlation between (the residuals of) different items on the off diagonals, and the difference (between simulated and real data) in the correlation between consecutive residuals of the same item on the diagonals (Table I).

The correlation plot showed quite a strong correlation among some of the items, specifically, between items 1, 5 and 6, items 7 and 11 (Fig. 5) and also between items 2 and 3, corresponding to different domains in the EXACT® questionnaire (Table I). Additionally, a relatively high correlation was also observed between two items from different domains (Table I), specifically item 12 and 13 (Fig. 5).

**Fig. 6:**
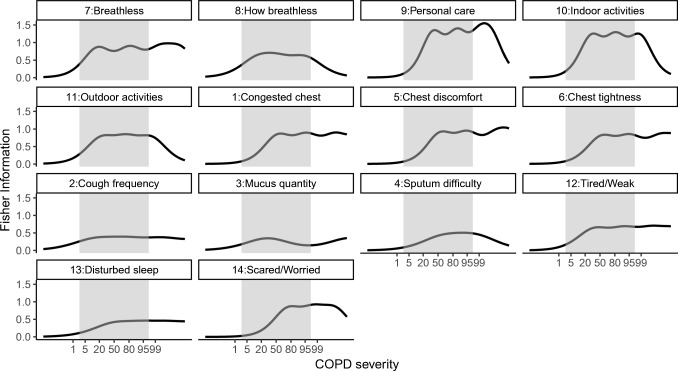
The Fisher information content per item, plotted against chronic obstructive pulmonary disease (COPD) severity, represented in percentiles of the data (*e.g.* 50 indicates a typical subject from this population). Grey area is the 95% confidence interval around the baseline disease severity. More detailed item descriptions are given in Table I.

**Fig. 7:**
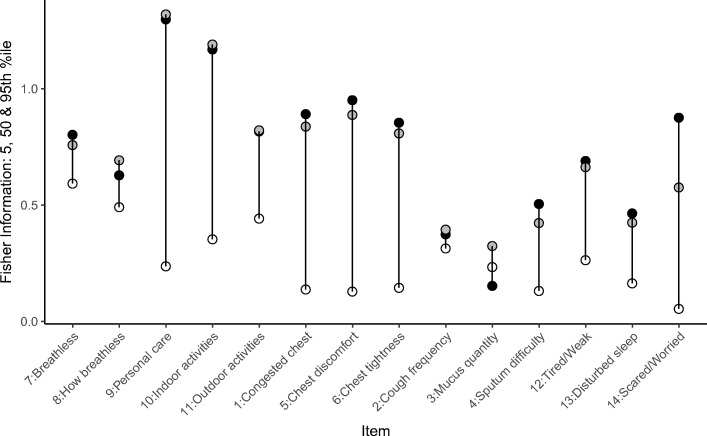
The Fisher information content for the ‘healthier’ (5th percentile, white circles), ‘typical’ (50th percentile, grey circles) and ‘sicker’ (95th percentile, black circles) parts of the studied population, for each item (Table I).

The items providing the most information in characterising the typical patient (*i.e.* patient with the typical disease severity) at baseline were ‘Breathless—personal care’ and ‘Breathless—indoor activities’ (Fig. 6). Some other items, however, proved to be less informative (*e.g.* ‘Sputum difficulty’) (Fig. 6). Also, in general, all items (except item 3, ‘Mucus quantity’) appeared to be more informative for the ‘sicker’, or a typical subject from the studied population, compared to the ‘healthier’ part of the population (Fig. 7). The item characteristic curves (ICC) for all 14 items are shown in Fig. S6 (supplementary material), and the values of the ICC parameters are reported in Table S2.

**Fig. 8:**
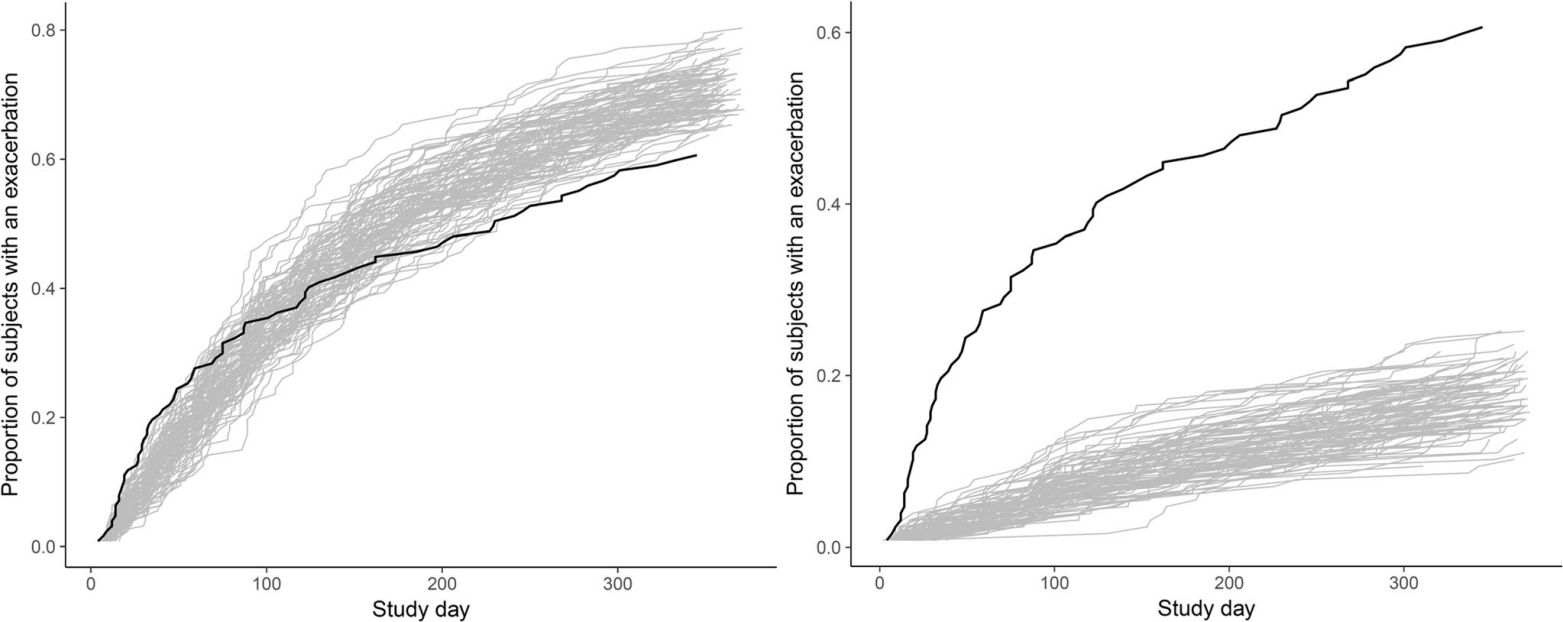
The cumulative proportion of participants who already had an exacerbation, from a model with the Markov elements (left), and a model without the Markov elements (right). Black lines represent data, and grey simulations (*n* = 100).

A comparison of model-simulated and observed cumulative proportion of subjects who already had a symptom-defined exacerbation event, from a model with the Markov elements and a model without the Markov elements is shown in Fig. 8 and indicated that the addition of the Markov elements improved model performance.

## DISCUSSION

A combination of an item response theory model and 14 item-specific longitudinal Markov models was successfully developed for the first time to our knowledge. This integrated modelling approach proved to be able to describe frequently collected and therefore correlated composite score data, as was exemplified here using daily EXACT® patient reported outcome data from patients with COPD receiving standard of care only.

The importance of developing a model, where IRT is used together with the longitudinal Markov elements, is twofold. Firstly, using the IRT methodology has several advantages over the total-score approach. For example, as it uses all individual item scores and not just a total score, it prevents information loss, which might otherwise result in model misspecification. Additionally, it does not ignore the categorical nature of the data, which occurs when modelling the total score as a single continuous variable. Secondly, by including the longitudinal Markov elements (on an item level), the developed model also provides a way to describe the dependence between frequently collected longitudinal data such as those obtained nowadays from patient-reported diaries which are now filled in at home, using an electronic device.

The mean equilibrium time was estimated longer than a day (1.2 days at start of the study, and 5.1 days at the end of the study, Table S2), and a clear misfit of the model without the Markov elements was observed (Fig. 8, Fig. S1), which both confirmed that the addition of the Markov elements was needed. The values of the MET parameter estimates also show that the variability in patients reporting outcomes was decreasing with time on study, since a higher MET value indicates fewer transitions, *i.e.* more stable scores. This might be because when a patient is recruited to a study, they might be more compliant with their medications and have more healthcare interactions.

The graphical evaluation of the model showed that the final model can describe the data adequately. More specifically, the model was able to describe the proportions of item-scores (Fig. 2 and Fig. S3a-d), the proportions of transitions between previous and current item-scores (Fig. 3), and also the total scores (Fig. 4). There were some underpredictions in the situation when an item score would not change from 1 (Fig. 2), and also in the median total scores (Fig. 4); however, these misspecifications were not that apparent, with the model adequately capturing the time trends and the 95% prediction intervals, and none of the additional changes to the model that were tested provided an improvement.

When correlations between items were investigated, it was confirmed that the grouping of the items in the COPD disease domains (as captured in the EXACT® questionnaire) was appropriate, since the correlation between items 1, 5 and 6; items 7 and 11; and items 2 and 3 (belonging to the chest domain, breathlessness domain and cough and sputum domain, respectively, Table I) were much stronger than between other items (Fig. 5). Additionally, although not belonging to the same domain, items 12 and 13 also showed to be correlated, which can be expected, given their description—*i.e.* ‘Tired or weak’, and ‘Disturbed sleep’, respectively (Table I). Since there were items belonging to obvious domains, one could argue that several latent variables should be included in the model; however, in our example, this was not possible due to too few items belonging to separate domains.

Since visual diagnostics confirmed that the model could describe the data satisfactorily, *i.e.* there were no major misspecifications, further potential improvements to the model were not investigated. For example, we used minimal Markov models, meaning that the MET was assumed to not differ between compartments, and a dropout model was also not investigated. Additionally, we did not evaluate any of the available covariates in this work, nevertheless, the model was able to describe the data when stratified by gender (Fig. S4) and also when plotted against age (Fig. S5), indicating that these covariates might not provide an improvement to the fit. Another potential limitation might be that we did not have information on whether the patients were at any time admitted to a hospital and treated with another drug (not standard of care), which may affect their disease progression.

In our model, continuous time Markov models were used, where, in contrast with discrete time Markov models, the probability of transition can change according to the time difference between two consecutive observations. Therefore, if a subject had missing observations for a study day, this did not affect the fit.

When the final model’s ability to predict symptom-defined exacerbations was tested, the model could predict the general trend of the cumulative proportions of the subjects with an exacerbation; however, some overprediction was observed when the study day was greater than approximately 180 days (Fig. 8), which might be due to fewer study participants continuing to report their symptoms. However, the model with the same structure without the Markov elements greatly underpredicted the symptom-defined exacerbations even from the beginning of the study. The poor performance of the model to predict symptom-defined exacerbations without the Markov elements can be understood from the definition of such exacerbation, *i.e.* the total score had to be increased for at least two or three consecutive days. As the variability in transitions between scores was exaggerated in the model without the Markov elements (Fig. S1), a stable increase in a score did not occur; hence, a symptom-defined exacerbation was not defined.

Future work could include adding a treatment effect to the model, and although the model was developed using EXACT® data, it could also be applied to daily data from other questionnaires. With a treatment effect added to the model, the model could be utilised for simulation of study designs in terms of evaluating treatment effects, sample size and duration. Additionally, the effect of patients’ characteristics on response over time, especially important in early drug development, could also be investigated and perhaps help shorten proof-of-concept studies.

### Conclusion

A longitudinal mixed-effects IRT model with Markov elements was developed for the first time to our knowledge and applied to real data from an observational study. The model was able to handle both composite scores and frequent observations, which was exemplified in this analysis using COPD item score and symptom-defined exacerbation data from the EXACT® questionnaire. The developed model also showed it could serve as a platform model for predicting symptom-defined exacerbations.

## Electronic Supplementary Material

ESM 1 (PDF 1.25 mb)

## References

[1] Ramsey SD, Hobbs FD (2006). Chronic obstructive pulmonary disease, risk factors, and outcome trials: comparisons with cardiovascular disease. Proc Am Thorac Soc.

[2] Wedzicha JA, Donaldson GC (2003). Exacerbations of chronic obstructive pulmonary disease. Respir Care.

[3] Donaldson GC, Seemungal TA, Patel IS, Lloyd-Owen SJ, Wilkinson TM, Wedzicha JA (2003). Longitudinal changes in the nature, severity and frequency of COPD exacerbations. Eur Respir J.

[4] EMA. European Medicines Agency. Draft qualification opinion of qualification of exacerbations of chronic pulmonary disease tool (EXACT), and EXACT-respiratory symptoms measure (E-RS) for evaluating treatment outcomes in clinical trials in COPD: http://www.ema.europa.eu/docs/en_GB/document_library/Regulatory_and_procedural_guideline/2015/04/WC500185442.pdf. Last Accessed 20 June 2017.

[5] Murray LT, Leidy NK (2018). The short-term impact of symptom-defined COPD exacerbation recovery on health status and lung function. Chronic Obstr Pulm Dis.

[6] FDA. Food and Drug Administration (FDA) US Department of Health and Human Services Center for Drug Evaluation and Research (CDER). Qualication of exacerbations of chronic pulmonary disease tool for measurement of symptoms of acute bacterial exacerbation of chronic bronchitis in patients with chronic obstructive pulmonary disease. Draft guidance; http://www.fda.gov/downloads/Drugs/GuidanceComplianceRegulatoryInformation/Guidances/UCM380961.pdf. Last Accessed 13 July 2018.

[7] Donaldson GC, Hurst JR, Smith CJ, Hubbard RB, Wedzicha JA (2010). Increased risk of myocardial infarction and stroke following exacerbation of COPD. Chest..

[8] Seemungal TA, Donaldson GC, Paul EA, Bestall JC, Jeffries DJ, Wedzicha JA (1998). Effect of exacerbation on quality of life in patients with chronic obstructive pulmonary disease. Am J Respir Crit Care Med.

[9] Soriano JB, Abajobir AA, Abate KH, Abera SF, Agrawal A, Ahmed MB, Aichour AN, Aichour I, Aichour MTE, Alam K, Alam N, Alkaabi JM, al-Maskari F, Alvis-Guzman N, Amberbir A, Amoako YA, Ansha MG, Antó JM, Asayesh H, Atey TM, Avokpaho EFGA, Barac A, Basu S, Bedi N, Bensenor IM, Berhane A, Beyene AS, Bhutta ZA, Biryukov S, Boneya DJ, Brauer M, Carpenter DO, Casey D, Christopher DJ, Dandona L, Dandona R, Dharmaratne SD, Do HP, Fischer F, Gebrehiwot TT, Geleto A, Ghoshal AG, Gillum RF, Ginawi IAM, Gupta V, Hay SI, Hedayati MT, Horita N, Hosgood HD, Jakovljevic M(M)B, James SL, Jonas JB, Kasaeian A, Khader YS, Khalil IA, Khan EA, Khang YH, Khubchandani J, Knibbs LD, Kosen S, Koul PA, Kumar GA, Leshargie CT, Liang X, el Razek HMA, Majeed A, Malta DC, Manhertz T, Marquez N, Mehari A, Mensah GA, Miller TR, Mohammad KA, Mohammed KE, Mohammed S, Mokdad AH, Naghavi M, Nguyen CT, Nguyen G, le Nguyen Q, Nguyen TH, Ningrum DNA, Nong VM, Obi JI, Odeyemi YE, Ogbo FA, Oren E, PA M, Park EK, Patton GC, Paulson K, Qorbani M, Quansah R, Rafay A, Rahman MHU, Rai RK, Rawaf S, Reinig N, Safiri S, Sarmiento-Suarez R, Sartorius B, Savic M, Sawhney M, Shigematsu M, Smith M, Tadese F, Thurston GD, Topor-Madry R, Tran BX, Ukwaja KN, van Boven JFM, Vlassov VV, Vollset SE, Wan X, Werdecker A, Hanson SW, Yano Y, Yimam HH, Yonemoto N, Yu C, Zaidi Z, el Sayed Zaki M, Lopez AD, Murray CJL, Vos T (2017). Global, regional, and national deaths, prevalence, disability-adjusted life years, and years lived with disability for chronic obstructive pulmonary disease and asthma, 1990–2015: a systematic analysis for the Global Burden of Disease Study 2015. Lancet Respir Med.

[10] Rabe KF, Watz H (2017). Chronic obstructive pulmonary disease. Lancet..

[11] Prince MJ, Wu F, Guo Y, Gutierrez Robledo LM, O'Donnell M, Sullivan R, Yusuf S (2015). The burden of disease in older people and implications for health policy and practice. Lancet..

[12] Lozano R, Naghavi M, Foreman K, Lim S, Shibuya K, Aboyans V, Abraham J, Adair T, Aggarwal R, Ahn SY, AlMazroa MA, Alvarado M, Anderson HR, Anderson LM, Andrews KG, Atkinson C, Baddour LM, Barker-Collo S, Bartels DH, Bell ML, Benjamin EJ, Bennett D, Bhalla K, Bikbov B, Abdulhak AB, Birbeck G, Blyth F, Bolliger I, Boufous S, Bucello C, Burch M, Burney P, Carapetis J, Chen H, Chou D, Chugh SS, Coffeng LE, Colan SD, Colquhoun S, Colson KE, Condon J, Connor MD, Cooper LT, Corriere M, Cortinovis M, de Vaccaro KC, Couser W, Cowie BC, Criqui MH, Cross M, Dabhadkar KC, Dahodwala N, de Leo D, Degenhardt L, Delossantos A, Denenberg J, Des Jarlais DC, Dharmaratne SD, Dorsey ER, Driscoll T, Duber H, Ebel B, Erwin PJ, Espindola P, Ezzati M, Feigin V, Flaxman AD, Forouzanfar MH, Fowkes FGR, Franklin R, Fransen M, Freeman MK, Gabriel SE, Gakidou E, Gaspari F, Gillum RF, Gonzalez-Medina D, Halasa YA, Haring D, Harrison JE, Havmoeller R, Hay RJ, Hoen B, Hotez PJ, Hoy D, Jacobsen KH, James SL, Jasrasaria R, Jayaraman S, Johns N, Karthikeyan G, Kassebaum N, Keren A, Khoo JP, Knowlton LM, Kobusingye O, Koranteng A, Krishnamurthi R, Lipnick M, Lipshultz SE, Ohno SL, Mabweijano J, MacIntyre MF, Mallinger L, March L, Marks GB, Marks R, Matsumori A, Matzopoulos R, Mayosi BM, McAnulty JH, McDermott MM, McGrath J, Memish ZA, Mensah GA, Merriman TR, Michaud C, Miller M, Miller TR, Mock C, Mocumbi AO, Mokdad AA, Moran A, Mulholland K, Nair MN, Naldi L, Narayan KMV, Nasseri K, Norman P, O'Donnell M, Omer SB, Ortblad K, Osborne R, Ozgediz D, Pahari B, Pandian JD, Rivero AP, Padilla RP, Perez-Ruiz F, Perico N, Phillips D, Pierce K, Pope CA, Porrini E, Pourmalek F, Raju M, Ranganathan D, Rehm JT, Rein DB, Remuzzi G, Rivara FP, Roberts T, de León FR, Rosenfeld LC, Rushton L, Sacco RL, Salomon JA, Sampson U, Sanman E, Schwebel DC, Segui-Gomez M, Shepard DS, Singh D, Singleton J, Sliwa K, Smith E, Steer A, Taylor JA, Thomas B, Tleyjeh IM, Towbin JA, Truelsen T, Undurraga EA, Venketasubramanian N, Vijayakumar L, Vos T, Wagner GR, Wang M, Wang W, Watt K, Weinstock MA, Weintraub R, Wilkinson JD, Woolf AD, Wulf S, Yeh PH, Yip P, Zabetian A, Zheng ZJ, Lopez AD, Murray CJL (2012). Global and regional mortality from 235 causes of death for 20 age groups in 1990 and 2010: a systematic analysis for the Global Burden of Disease Study 2010. Lancet..

[13] Spencer M, Briggs AH, Grossman RF, Rance L (2005). Development of an economic model to assess the cost effectiveness of treatment interventions for chronic obstructive pulmonary disease. PharmacoEconomics..

[14] Borg S, Ericsson A, Wedzicha J, Gulsvik A, Lundback B, Donaldson GC (2004). A computer simulation model of the natural history and economic impact of chronic obstructive pulmonary disease. Value Health.

[15] Slejko JF, Willke RJ, Ribbing J, Milligan P (2016). Translating pharmacometrics to a pharmacoeconomic model of COPD. Value Health.

[16] Hoogendoorn M, Rutten-van Molken MP, Hoogenveen RT, Al MJ, Feenstra TL (2011). Developing and applying a stochastic dynamic population model for chronic obstructive pulmonary disease. Value Health.

[17] Menn P, Leidl R, Holle R (2012). A lifetime Markov model for the economic evaluation of chronic obstructive pulmonary disease. PharmacoEconomics..

[18] Bertens LC, Reitsma JB, Moons KG, van Mourik Y, Lammers JW, Broekhuizen BD (2013). Development and validation of a model to predict the risk of exacerbations in chronic obstructive pulmonary disease. Int J Chron Obstruct Pulmon Dis.

[19] Montserrat-Capdevila J, Godoy P, Marsal JR, Barbe F (2015). Predictive model of hospital admission for COPD exacerbation. Respir Care.

[20] Exuzides A, Colby C, Briggs AH, Lomas DA, Rutten-van Molken M, Tabberer M (2017). Statistical modeling of disease progression for chronic obstructive pulmonary disease using data from the ECLIPSE study. Med Decis Making.

[21] Tabberer M, Gonzalez-McQuire S, Muellerova H, Briggs AH, Rutten-van Molken M, Chambers M (2017). Development of a conceptual model of disease progression for use in economic modeling of chronic obstructive pulmonary disease. Med Decis Making.

[22] Briggs AH, Baker T, Risebrough NA, Chambers M, Gonzalez-McQuire S, Ismaila AS, Exuzides A, Colby C, Tabberer M, Muellerova H, Locantore N, Rutten van Mölken MPMH, Lomas DA (2017). Development of the Galaxy Chronic Obstructive Pulmonary Disease (COPD) model using data from ECLIPSE: internal validation of a linked-equations cohort model. Med Decis Making.

[23] Basch E (2010). The missing voice of patients in drug-safety reporting. N Engl J Med.

[24] McKenna SP (2011). Measuring patient-reported outcomes: moving beyond misplaced common sense to hard science. BMC Med.

[25] FDA (2006). Guidance for industry: patient-reported outcome measures: use in medical product development to support labeling claims: draft guidance. Health Qual Life Outcomes.

[26] Blanchin M, Hardouin JB, Le Neel T, Kubis G, Blanchard C, Mirallie E (2011). Comparison of CTT and Rasch-based approaches for the analysis of longitudinal patient reported outcomes. Stat Med.

[27] Nguyen TH, Han HR, Kim MT, Chan KS (2014). An introduction to item response theory for patient-reported outcome measurement. Patient.

[28] Ueckert S, Plan EL, Ito K, Karlsson MO, Corrigan B, Hooker AC (2014). Improved utilization of ADAS-cog assessment data through item response theory based pharmacometric modeling. Pharm Res.

[29] Novakovic AM, Krekels EH, Munafo A, Ueckert S, Karlsson MO (2017). Application of item response theory to modeling of expanded disability status scale in multiple sclerosis. AAPS J.

[30] Krekels EHJ, Novakovic AM, Vermeulen AM, Friberg LE, Karlsson MO (2017). Item response theory to quantify longitudinal placebo and paliperidone effects on PANSS scores in schizophrenia. CPT Pharmacometrics Syst Pharmacol.

[31] Buatois S, Retout S, Frey N, Ueckert S (2017). Item response theory as an efficient tool to describe a heterogeneous clinical rating scale in de novo idiopathic Parkinson’s disease patients. Pharm Res.

[32] Gottipati G, Karlsson MO, Plan EL (2017). Modeling a composite score in Parkinson’s disease using item response theory. AAPS J.

[33] Valitalo PA, van Dijk M, Krekels EH, Gibbins S, Simons SH, Tibboel D (2016). Pain and distress caused by endotracheal suctioning in neonates is better quantified by behavioural than physiological items: a comparison based on item response theory modelling. Pain..

[34] Chae D, Park K (2018). An item response theory based integrated model of headache, nausea, photophobia, and phonophobia in migraine patients. J Pharmacokinet Pharmacodyn.

[35] Vandemeulebroecke M, Bornkamp B, Krahnke T, Mielke J, Monsch A, Quarg P (2017). A longitudinal item response theory model to characterize cognition over time in elderly subjects. CPT Pharmacometrics Syst Pharmacol.

[36] Bourne S, Cohet C, Kim V, Barton A, Tuck A, Aris E, Mesia-Vela S, Devaster JM, Ballou WR, Clarke S, Wilkinson T (2014). Acute Exacerbation and Respiratory InfectionS in COPD (AERIS): protocol for a prospective, observational cohort study. BMJ Open.

[37] Wilkinson TMA, Aris E, Bourne S, Clarke SC, Peeters M, Pascal TG, Schoonbroodt S, Tuck AC, Kim V, Ostridge K, Staples KJ, Williams N, Williams A, Wootton S, Devaster JM, AERIS Study Group (2017). A prospective, observational cohort study of the seasonal dynamics of airway pathogens in the aetiology of exacerbations in COPD. Thorax..

[38] Leidy NK, Murray LT, Monz BU, Nelsen L, Goldman M, Jones PW, Dansie EJ, Sethi S (2014). Measuring respiratory symptoms of COPD: performance of the EXACT- Respiratory Symptoms Tool (E-RS) in three clinical trials. Respir Res.

[39] Leidy NK, Murray LT (2013). Patient-reported outcome (PRO) measures for clinical trials of COPD: the EXACT and E-RS. Copd..

[40] GlaxoSmithKline. Contribution of infectious pathogens to acute respiratory illness in adults and elderly https://www.gsk-clinicalstudyregister.com/search/?search_terms=NCT01360398. Last accessed 13 July 2018.

[41] EXACT-PRO initiative. The Exacerbations of Chronic Pulmonary Disease Tool (EXACT). Patient-Reported Outcome (PRO). User manual (Version 7.0) www.ema.europa.eu/docs/en_GB/document_library/Other/2015/04/WC500185444.pdf. Last Accessed 12 July 2018.

[42] DeMars C. Item response theory: Oxford University Press; 2010.

[43] Schindler E, Karlsson MO (2017). A minimal continuous-time Markov pharmacometric model. AAPS J.

[44] Boeckmann AJ, Beal SL, Sheiner LB (1999). NONMEM users guide.

[45] R Core Team. R: A language and environment for statistical computing. R Foundation for Statistical Computing, Vienna, Austria. URL https://www.R-project.org/. 2016. Access July 2016

[46] Lindbom Lars, Ribbing Jakob, Jonsson E.Niclas (2004). Perl-speaks-NONMEM (PsN)—a Perl module for NONMEM related programming. Computer Methods and Programs in Biomedicine.

[47] Wickham H, Francois R. dplyr: a grammar of data manipulation. R package version 0.5.0. https://CRAN.R-project.org/package=dplyr. 2016. Access July 2016

[48] Jonsson EN, Karlsson MO (1999). Xpose--an S-PLUS based population pharmacokinetic/pharmacodynamic model building aid for NONMEM. Comput Methods Prog Biomed.

[49] Wickham H (2010). ggplot2: elegant graphics for data analysis.

[50] Lehmann EL, Casella G (1998). Theory of point estimation.

[51] Global Initiative for Chronic Obstructive Lung Disease. Global strategy for the diagnosis, management, and prevention of COPD (revised 2011). Report. 2011.

