# Supplementary material for: A Novel Method for Analysing Frequent Observations from Questionnaires in Order to Model Patient-Reported Outcomes: Application to EXACT® Daily Diary Data from COPD Patients
Source: AAPS J. 2019 Apr 26;21(4):60. doi: 10.1208/s12248-019-0319-9 (PMC6486532; doi:10.1208/s12248-019-0319-9)
Supplement: Supplementary file 1 — (PDF 1.25 mb) [file 12248_2019_319_MOESM1_ESM.pdf]

Supplementary material

Model code

```
$SIZES      MAXFCN=100000000
$PROBLEM    COPD PRO data modelling: IRT+ODEs
$INPUT      ID ITEM DAY VISIT DV AGE FEMALE SUMSCORE TOTAL
            EVID CMT AMT TIME
; time starts at 0
; items 3, 8, 10, 11, 14 have DV 0-3; and the rest 0-4
; sumscore is a sum of item scores on a certain day
; total is a flag indicating that item 99 is total score

$DATA      prodata.csv IGNORE=@
           IGNORE=(TOTAL==1)   ; ignore total score rows

$SUBROUTINE ADVAN6 TOL=6
$MODEL      NCOMPS=5
            COMP=SCR0 ;SCORE=0
            COMP=SCR1 ;SCORE=1
            COMP=SCR2 ;SCORE=2
            COMP=SCR3 ;SCORE=3
            COMP=SCR4 ;SCORE=4

$PK

; --------- (re)set the PDV -------------------------
IF(NEWIND.NE.2.OR.TIME<0.1) THEN     ; first row of a subject or an item
 IF(EVID==0) TMPDV=DV ; first PDV=DV
 NREC=1
 OLDTIME=TIME
ENDIF

XNRC = NREC
IF(TIME>OLDTIME) NREC=NREC+1

IF(EVID==0) PDV=TMPDV

;------------  item parameter selection ---------------------
IF(ITEM==1) THEN
 DIS   = THETA(1)
 DIF1  = THETA(2)
 DIF2  = THETA(3)
 DIF3  = THETA(4)
 DIF4  = THETA(5)
ENDIF

IF(ITEM==2) THEN
 DIS   = THETA(6)
 DIF1  = THETA(7)
 DIF2  = THETA(8)
 DIF3  = THETA(9)
 DIF4  = THETA(10)
ENDIF

IF(ITEM==3) THEN
 DIS   = THETA(11)
 DIF1  = THETA(12)
 DIF2  = THETA(13)
 DIF3  = THETA(14)
 DIF4  = THETA(15)  ; no need, only 0-3
```

```
ENDIF

IF(ITEM==4) THEN
 DIS   = THETA(16)
 DIF1  = THETA(17)
 DIF2  = THETA(18)
 DIF3  = THETA(19)
 DIF4  = THETA(20)
ENDIF

IF(ITEM==5) THEN
 DIS   = THETA(21)
 DIF1  = THETA(22)
 DIF2  = THETA(23)
 DIF3  = THETA(24)
 DIF4  = THETA(25)
ENDIF

IF(ITEM==6) THEN
 DIS   = THETA(26)
 DIF1  = THETA(27)
 DIF2  = THETA(28)
 DIF3  = THETA(29)
 DIF4  = THETA(30)
ENDIF

IF(ITEM==7) THEN
 DIS   = THETA(31)
 DIF1  = THETA(32)
 DIF2  = THETA(33)
 DIF3  = THETA(34)
 DIF4  = THETA(35)
ENDIF

IF(ITEM==8) THEN
 DIS   = THETA(36)
 DIF1  = THETA(37)
 DIF2  = THETA(38)
 DIF3  = THETA(39)
 DIF4  = THETA(40)   ; no need, only 0-3
ENDIF

IF(ITEM==9) THEN
 DIS   = THETA(41)
 DIF1  = THETA(42)
 DIF2  = THETA(43)
 DIF3  = THETA(44)
 DIF4  = THETA(45)
ENDIF

IF(ITEM==10) THEN
 DIS   = THETA(46)
 DIF1  = THETA(47)
 DIF2  = THETA(48)
 DIF3  = THETA(49)
 DIF4  = THETA(50)    ; no need, only 0-3
ENDIF

IF(ITEM==11) THEN
 DIS   = THETA(51)
 DIF1  = THETA(52)
```

```
 DIF2  = THETA(53)
 DIF3  = THETA(54)
 DIF4  = THETA(55)   ; no need, only 0-3
ENDIF

IF(ITEM==12) THEN
 DIS   = THETA(56)
 DIF1  = THETA(57)
 DIF2  = THETA(58)
 DIF3  = THETA(59)
 DIF4  = THETA(60)
ENDIF

IF(ITEM==13) THEN
 DIS   = THETA(61)
 DIF1  = THETA(62)
 DIF2  = THETA(63)
 DIF3  = THETA(64)
 DIF4  = THETA(65)
ENDIF

IF(ITEM==14) THEN
 DIS   = THETA(66)
 DIF1  = THETA(67)
 DIF2  = THETA(68)
 DIF3  = THETA(69)
 DIF4  = THETA(70)   ; no need, only 0-3
ENDIF


;-----------------  hidden variable model  -------------------
BASELINE=THETA(71)+ETA(1)
SLP=THETA(72)+ETA(2)
PSI=BASELINE+SLP*TIME      ; COPD disease severity


;----------  2 parameter logit model implementation (4/5 states) ------

;------ constrain different states to be >= than the previous -------
DIFS1 = DIF1
DIFS2 = DIFS1+DIF2
DIFS3 = DIFS2+DIF3
DIFS4 = DIFS3+DIF4
IF(ITEM==3.OR.ITEM==8.OR.ITEM==10.OR.ITEM==11.OR.ITEM==14) DIFS4 = 0

; ------ probabilities for Y greater than 1 etc -------------
PGE1 = EXP(DIS*(PSI-DIFS1))/(1+EXP(DIS*(PSI-DIFS1)))
PGE2 = EXP(DIS*(PSI-DIFS2))/(1+EXP(DIS*(PSI-DIFS2)))
PGE3 = EXP(DIS*(PSI-DIFS3))/(1+EXP(DIS*(PSI-DIFS3)))
PGE4 = EXP(DIS*(PSI-DIFS4))/(1+EXP(DIS*(PSI-DIFS4)))
IF(ITEM==3.OR.ITEM==8.OR.ITEM==10.OR.ITEM==11.OR.ITEM==14) PGE4 = 0

; ------ probabilities for Y=0 etc --------------------------
P0 = 1-PGE1
P1 = PGE1-PGE2
P2 = PGE2-PGE3
P3 = PGE3-PGE4
IF(ITEM==3.OR.ITEM==8.OR.ITEM==10.OR.ITEM==11.OR.ITEM==14) P3 = PGE3
P4 = PGE4
IF(ITEM==3.OR.ITEM==8.OR.ITEM==10.OR.ITEM==11.OR.ITEM==14) P4 = 0
```

```
;---------- mean residence/equilibrium time ------------------
MET = (THETA(73)*EXP(ETA(3)) * (TIME/364)) + (THETA(74)*EXP(ETA(4)) * (1 -
TIME/364))

IF(P0<1E-16)  P0 = 1E-16
IF(P1<1E-16)  P1 = 1E-16
IF(P2<1E-16)  P2 = 1E-16
IF(P3<1E-16)  P3 = 1E-16
IF(P4<1E-16)  P4 = 1E-16


;--------------    Markov model  -------------------
K01  = 1/(MET*(1+P0/P1))
K10  = K01 *P0/P1
K12  = 1/(MET*(1+P1/P2))
K21  = K12 *P1/P2
K23  = 1/(MET*(1+P2/P3))
K32  = K23 *P2/P3

K34  = 1/(MET*(1+P3/P4))
K43  = K34 *P3/P4
IF(ITEM==3.OR.ITEM==8.OR.ITEM==10.OR.ITEM==11.OR.ITEM==14) K34=0
IF(ITEM==3.OR.ITEM==8.OR.ITEM==10.OR.ITEM==11.OR.ITEM==14) K43=0


$DES

;-------- differential equations for a 5 state MM ------------
DADT(1) = - K01*A(1) + K10*A(2)
DADT(2) = - (K10+K12)*A(2) + K01*A(1) + K21*A(3)
DADT(3) = - (K21+K23)*A(3) + K12*A(2) + K32*A(4)
DADT(4) = - (K32+K34)*A(4) + K23*A(3) + K43*A(5)
DADT(5) = - K43*A(5) + K34*A(4)


$ERROR

IF(DV==0) Y = A(1)
IF(DV==1) Y = A(2)
IF(DV==2) Y = A(3)
IF(DV==3) Y = A(4)
IF(DV==4) Y = A(5)

IF(TIME<0.01.AND.DV==0) Y=P0
IF(TIME<0.01.AND.DV==1) Y=P1
IF(TIME<0.01.AND.DV==2) Y=P2
IF(TIME<0.01.AND.DV==3) Y=P3
IF(TIME<0.01.AND.DV==4) Y=P4

A1 = A(1)
A2 = A(2)
A3 = A(3)
A4 = A(4)
A5 = A(5)

IPRED = (A1*0)+(A2*1)+(A3*2)+(A4*3)+(A5*4)
IF(TIME<0.01) IPRED = (P0*0)+(P1*1)+(P2*2)+(P3*3)+(P4*4)
RES = DV - IPRED
```

```
;----- get XDV ------------------------------------
XDV=0
IF (EVID==0) XDV = PDV*10 + DV


;----- remember TIME, DV  --------------------------
OLDTIME=TIME
IF(EVID==0.AND.ITEM.NE.99) TMPDV = DV


$THETA  (0,1.8094) ; 1. DIS_i1
$THETA  0.109885 ; 2. DIF1_i1
$THETA  (0,1.59965,1000000) ; 3. DIF2_i1
$THETA  (0,1.6405,1000000) ; 4. DIF3_i1
$THETA  (0,1.53025,1000000) ; 5. DIF4_i1
$THETA  (0,1.16486) ; 6. DIS_i2
$THETA  -1.02817 ; 7. DIF1_i2
$THETA  (0,1.72943,1000000) ; 8. DIF2_i2
$THETA  (0,2.09474,1000000) ; 9. DIF3_i2
$THETA  (0,2.62226,1000000) ; 10. DIF4_i2
$THETA  (0,1.17671) ; 11. DIS_i3
$THETA  -0.490139 ; 12. DIF1_i3
$THETA  (0,4.6755,1000000) ; 13. DIF2_i3
$THETA  (0,2.57238,1000000) ; 14. DIF3_i3
$THETA  0 FIX ; 15. DIF4_i3
$THETA  (0,1.2651) ; 16. DIS_i4
$THETA  0.242375 ; 17. DIF1_i4
$THETA  (0,1.04137,1000000) ; 18. DIF2_i4
$THETA  (0,0.616718,1000000) ; 19. DIF3_i4
$THETA  (0,0.342467,1000000) ; 20. DIF4_i4
$THETA  (0,1.86908) ; 21. DIS_i5
$THETA  0.132537 ; 22. DIF1_i5
$THETA  (0,1.51235,1000000) ; 23. DIF2_i5
$THETA  (0,1.69262,1000000) ; 24. DIF3_i5
$THETA  (0,0.829534,1000000) ; 25. DIF4_i5
$THETA  (0,1.76933) ; 26. DIS_i6
$THETA  0.0889128 ; 27. DIF1_i6
$THETA  (0,1.5799,1000000) ; 28. DIF2_i6
$THETA  (0,1.76672,1000000) ; 29. DIF3_i6
$THETA  (0,1.26297,1000000) ; 30. DIF4_i6
$THETA  (0,1.83855) ; 31. DIS_i7
$THETA  -0.927622 ; 32. DIF1_i7
$THETA  (0,1.82299,1000000) ; 33. DIF2_i7
$THETA  (0,1.72984,1000000) ; 34. DIF3_i7
$THETA  (0,1.09852,1000000) ; 35. DIF4_i7
$THETA  (0,1.53297) ; 36. DIS_i8
$THETA  -0.999236 ; 37. DIF1_i8
$THETA  (0,0.977651,1000000) ; 38. DIF2_i8
$THETA  (0,1.63974,1000000) ; 39. DIF3_i8
$THETA  0 FIX ; 40. DIF4_i8
$THETA  (0,2.27268) ; 41. DIS_i9
$THETA  -0.284162 ; 42. DIF1_i9
$THETA  (0,1.3684,1000000) ; 43. DIF2_i9
$THETA  (0,1.28837,1000000) ; 44. DIF3_i9
$THETA  (0,0.585445,1000000) ; 45. DIF4_i9
$THETA  (0,2.18176) ; 46. DIS_i10
$THETA  -0.482569 ; 47. DIF1_i10
$THETA  (0,1.36381,1000000) ; 48. DIF2_i10
$THETA  (0,1.36594,1000000) ; 49. DIF3_i10
$THETA  0 FIX ; 50. DIF4_i10
$THETA  (0,1.72962) ; 51. DIS_i11
```

```
$THETA  -0.718094 ; 52. DIF1_i11
$THETA  (0,1.39328,1000000) ; 53. DIF2_i11
$THETA  (0,1.41983,1000000) ; 54. DIF3_i11
$THETA  0 FIX ; 55. DIF4_i11
$THETA  (0,1.56876) ; 56. DIS_i12
$THETA  -0.335125 ; 57. DIF1_i12
$THETA  (0,1.67839,1000000) ; 58. DIF2_i12
$THETA  (0,1.56633,1000000) ; 59. DIF3_i12
$THETA  (0,1.39995,1000000) ; 60. DIF4_i12
$THETA  (0,1.24578) ; 61. DIS_i13
$THETA  0.0128549 ; 62. DIF1_i13
$THETA  (0,1.57365,1000000) ; 63. DIF2_i13
$THETA  (0,1.47766,1000000) ; 64. DIF3_i13
$THETA  (0,1.707,1000000) ; 65. DIF4_i13
$THETA  (0,1.79806) ; 66. DIS_i14
$THETA  0.674351 ; 67. DIF1_i14
$THETA  (0,1.43087,1000000) ; 68. DIF2_i14
$THETA  (0,1.2145,1000000) ; 69. DIF3_i14
$THETA  0 FIX ; 70. DIF4_i14
$THETA  0 FIX ; 71. baseline
$THETA  1.94669E-05 ; 72. slope
$THETA  (0,5.07916) ; 73. MET_end
$THETA  (0,1.22606) ; 74. MET_start
$OMEGA  1  FIX  ; 1. iiv_baseline
$OMEGA  1.11642E-05  ; 2. iiv_slope
$OMEGA  1.56713  ; 3. iiv_metEnd
$OMEGA  0.451146  ; 4. iiv_metStart

$ESTIMATION MAXEVAL=9999 METHOD=1 LAPLACE LIKE PRINT=1 NSIG=2 NOABORT
            MSFO=msf112
$COVARIANCE
```

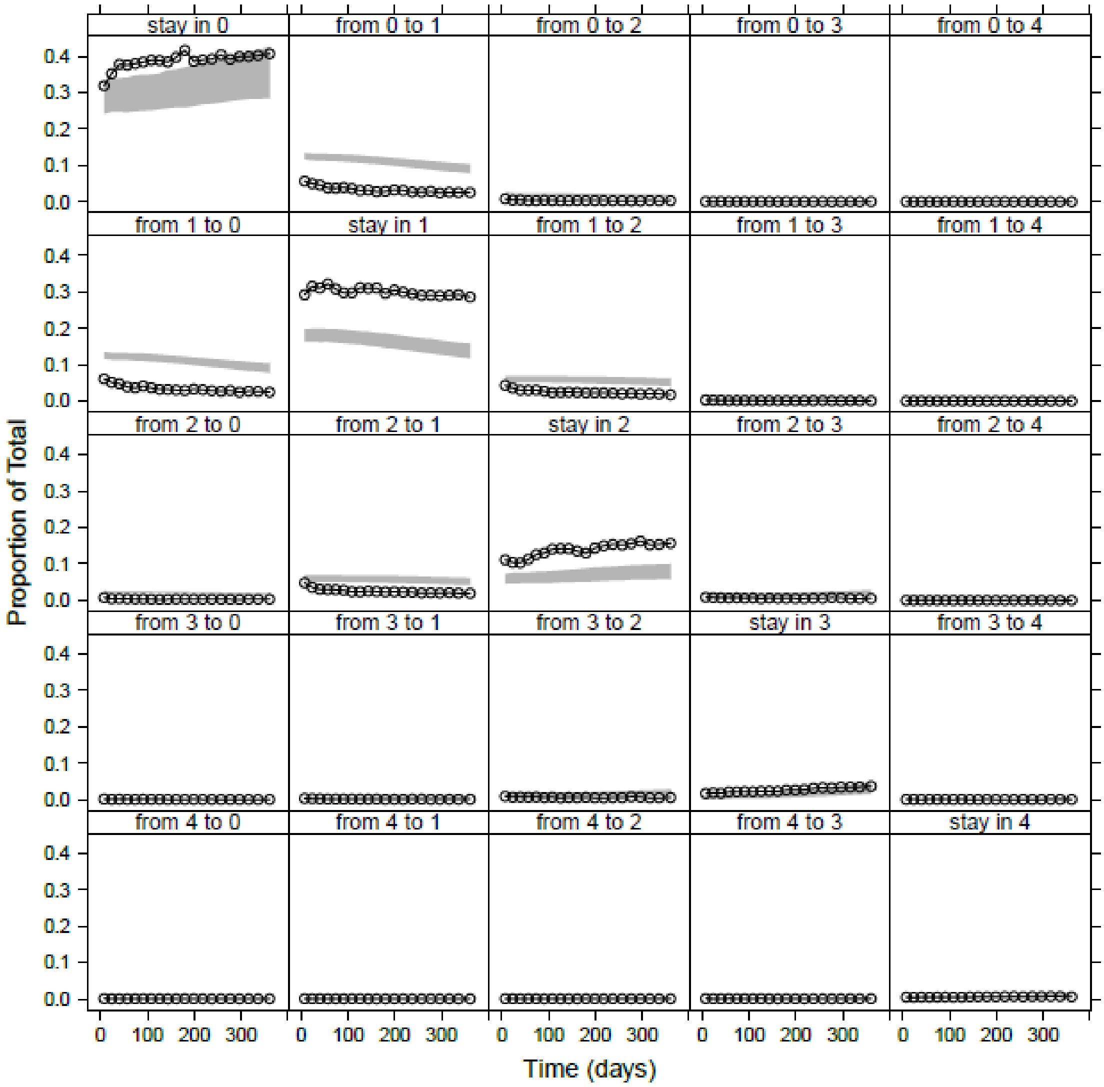

Figure S1: Visual predictive check for all 14 items, showing different proportions of observed transitions (black lines) with the corresponding 95% confidence intervals (grey areas) from 1,000 simulations using the model without Markov elements. Transitions are described in the panels.

Table S2: Final parameter estimates with uncertainty

	mean	SE
**1. DIS_i1**	1.81	0.19
**2. DIF1_i1**	0.11	0.12
**3. DIF2_i1**	1.60	0.13
**4. DIF3_i1**	1.64	0.16
**5. DIF4_i1**	1.53	0.31
**6. DIS_i2**	1.16	0.13
**7. DIF1_i2**	-1.03	0.23
**8. DIF2_i2**	1.73	0.21
**9. DIF3_i2**	2.09	0.23
**10. DIF4_i2**	2.62	0.45
**11. DIS_i3**	1.18	0.16
**12. DIF1_i3**	-0.49	0.20
**13. DIF2_i3**	4.68	0.55
**14. DIF3_i3**	2.57	0.43
**15. DIF4_i3**	0	na
**16. DIS_i4**	1.27	0.17
**17. DIF1_i4**	0.24	0.14
**18. DIF2_i4**	1.04	0.12
**19. DIF3_i4**	0.62	0.13
**20. DIF4_i4**	0.34	0.12
**21. DIS_i5**	1.87	0.17
**22. DIF1_i5**	0.13	0.12
**23. DIF2_i5**	1.51	0.13
**24. DIF3_i5**	1.69	0.14
**25. DIF4_i5**	0.83	0.14
**26. DIS_i6**	1.77	0.16
**27. DIF1_i6**	0.09	0.12
**28. DIF2_i6**	1.58	0.14
**29. DIF3_i6**	1.77	0.17
**30. DIF4_i6**	1.26	0.19
**31. DIS_i7**	1.84	0.15
**32. DIF1_i7**	-0.93	0.14
**33. DIF2_i7**	1.82	0.15
**34. DIF3_i7**	1.73	0.14
**35. DIF4_i7**	1.10	0.15
**36. DIS_i8**	1.53	0.16
**37. DIF1_i8**	-1.00	0.17
**38. DIF2_i8**	0.98	0.13
**39. DIF3_i8**	1.64	0.20
**40. DIF4_i8**	0	na
**41. DIS_i9**	2.27	0.22
**42. DIF1_i9**	-0.28	0.13
**43. DIF2_i9**	1.37	0.13
**44. DIF3_i9**	1.29	0.14
**45. DIF4_i9**	0.59	0.09
**46. DIS_i10**	2.18	0.20
**47. DIF1_i10**	-0.48	0.12
**48. DIF2_i10**	1.36	0.12

**49. DIF3_i10**	1.37	0.14
**50. DIF4_i10**	0	na
**51. DIS_i11**	1.73	0.18
**52. DIF1_i11**	-0.72	0.14
**53. DIF2_i11**	1.39	0.14
**54. DIF3_i11**	1.42	0.17
**55. DIF4_i11**	0	na
**56. DIS_i12**	1.57	0.14
**57. DIF1_i12**	-0.34	0.12
**58. DIF2_i12**	1.68	0.13
**59. DIF3_i12**	1.57	0.19
**60. DIF4_i12**	1.40	0.23
**61. DIS_i13**	1.25	0.14
**62. DIF1_i13**	0.01	0.15
**63. DIF2_i13**	1.57	0.16
**64. DIF3_i13**	1.48	0.21
**65. DIF4_i13**	1.71	0.33
**66. DIS_i14**	1.80	0.33
**67. DIF1_i14**	0.67	0.22
**68. DIF2_i14**	1.43	0.18
**69. DIF3_i14**	1.21	0.36
**70. DIF4_i14**	0	na
**71. baseline**	0	na
**72. slope (/day)**	1.95E-05	0.0002
**72. slope (/yr)**	0.007	0.08
**73. MET_end (/day)**	5.08	0.57
**74. MET_start (/day)**	1.23	0.07
**1. iiv_baseline***	1	na
**2. iiv_slope (/day)***	1.12E-05	2.96E-06
**2. iiv_slope (/yr)***	1.49	0.39
**3. iiv_metEnd***	1.57	0.22
**4. iiv_metStart***	0.45	0.11

*variance scale; DIS=discrimination parameter (a_j), DIF=difficulty parameter (b_j), SE=standard error from NONMEM covariance step, iiv=interindividual variability, na=not available.

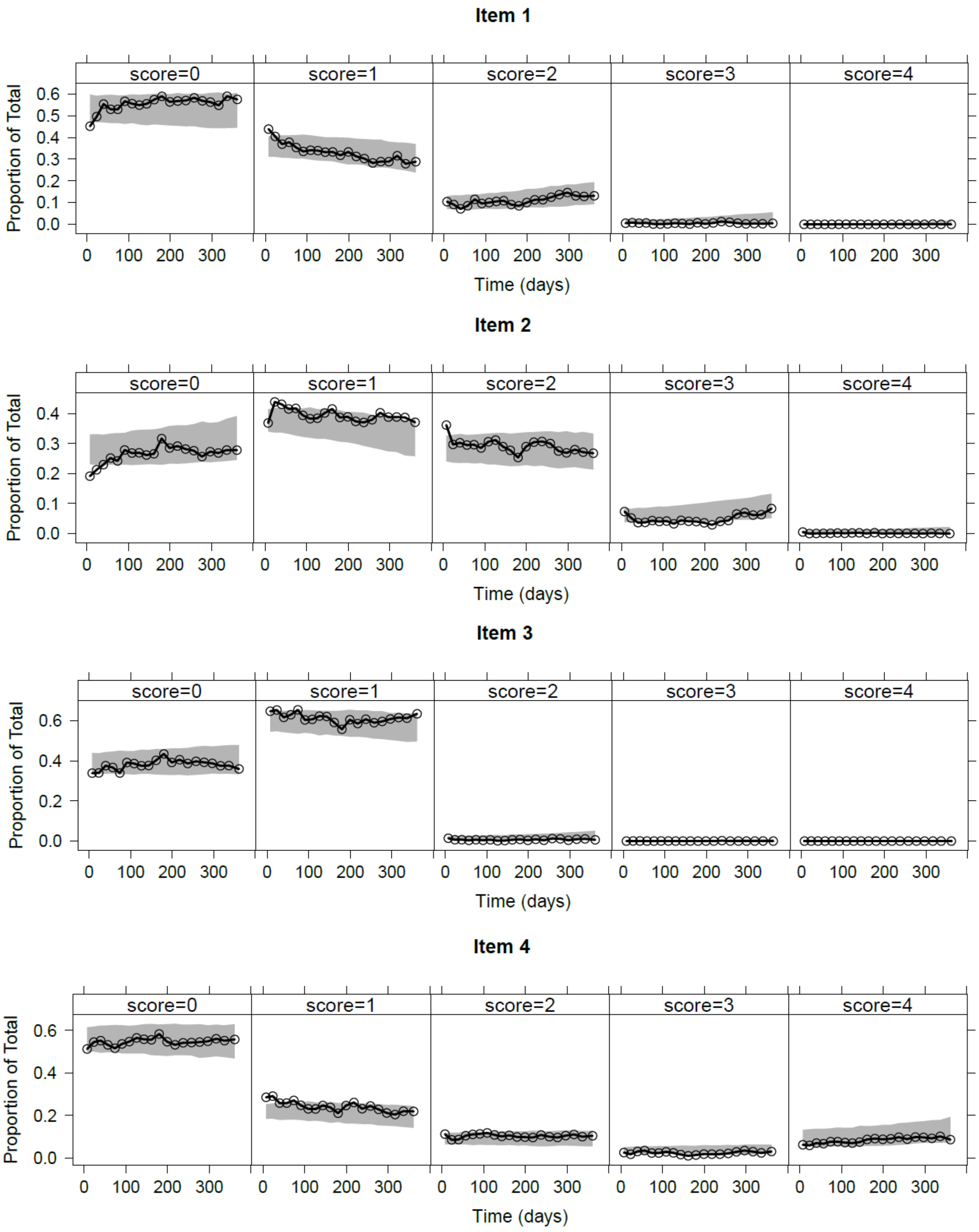

Figure S3a: Visual predictive check for the itemscores, stratified by items 1-4, showing different proportions of observations (black lines) with the corresponding 95% confidence interval (grey area) from 1,000 simulations.

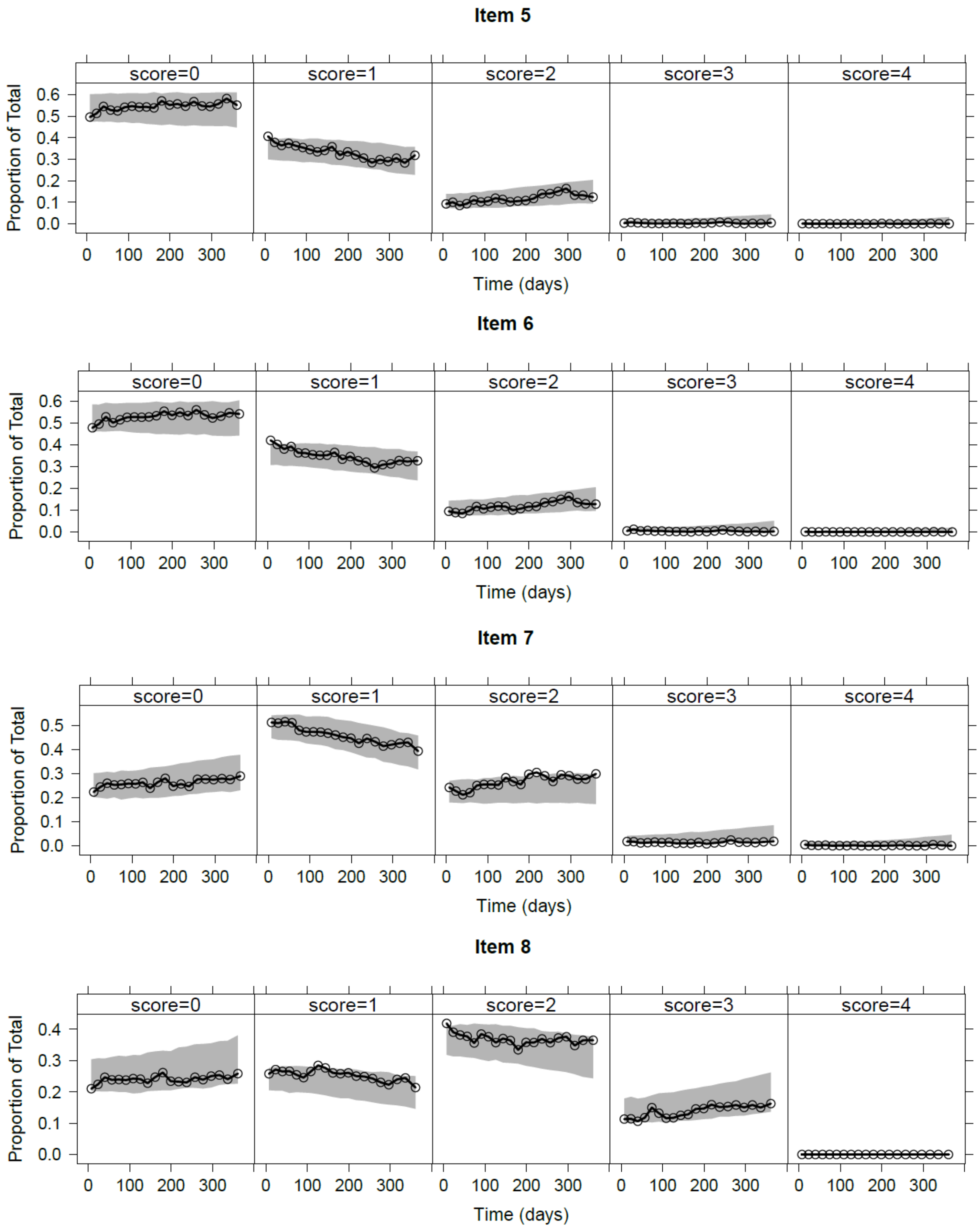

Figure S3b: Visual predictive check for the item scores, stratified by items 5-8, showing different proportions of observations (black lines) with the corresponding 95% confidence interval (grey area) from 1,000 simulations.

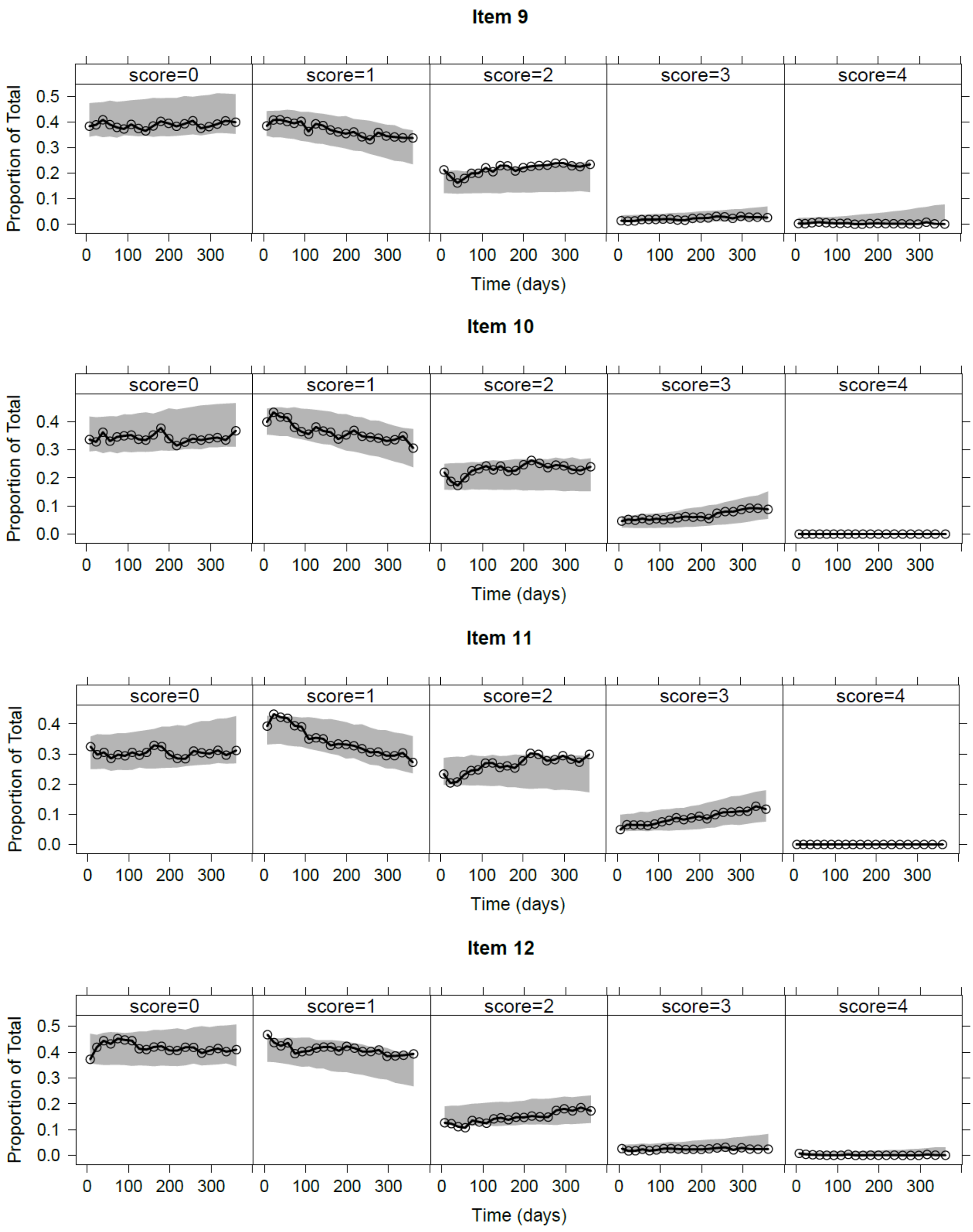

Figure S3c: Visual predictive check for the item scores, stratified by items 9-12, showing different proportions of observations (black lines) with the corresponding 95% confidence interval (grey area) from 1,000 simulations.

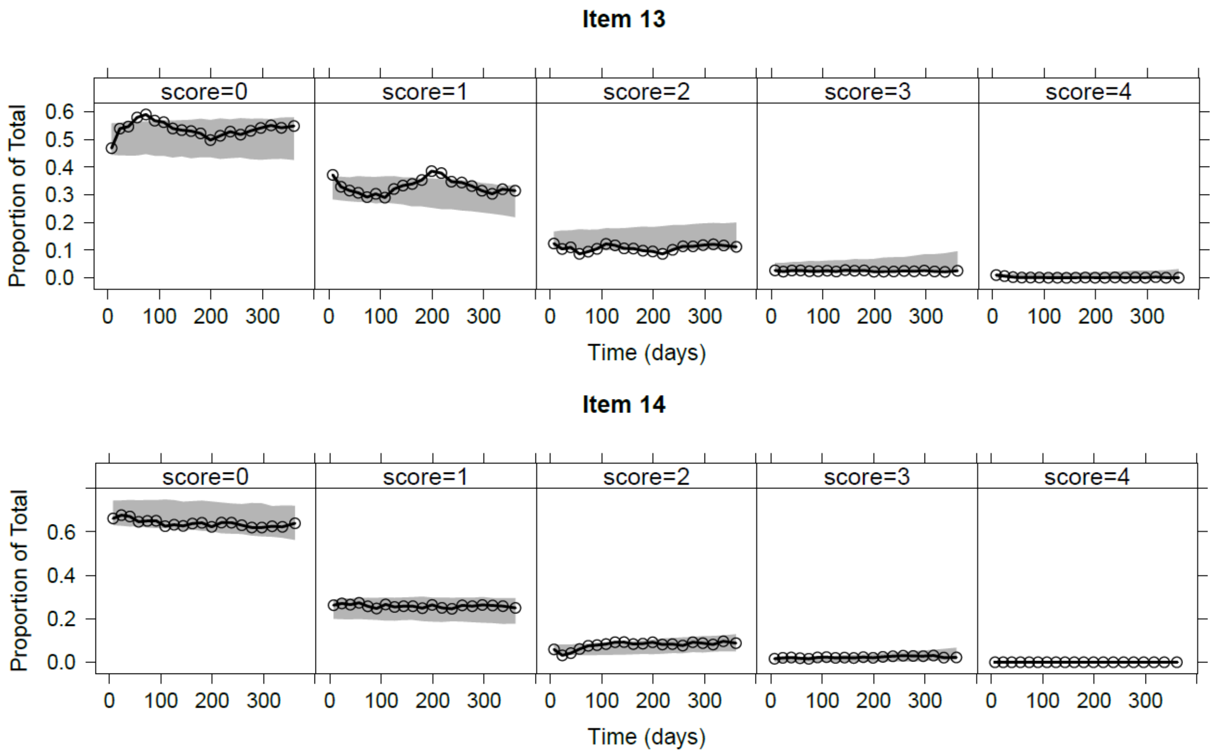

Figure S3d: Visual predictive check for the item scores, stratified by items 13-14, showing different proportions of observations (black lines) with the corresponding 95% confidence interval (grey area) from 1,000 simulations.

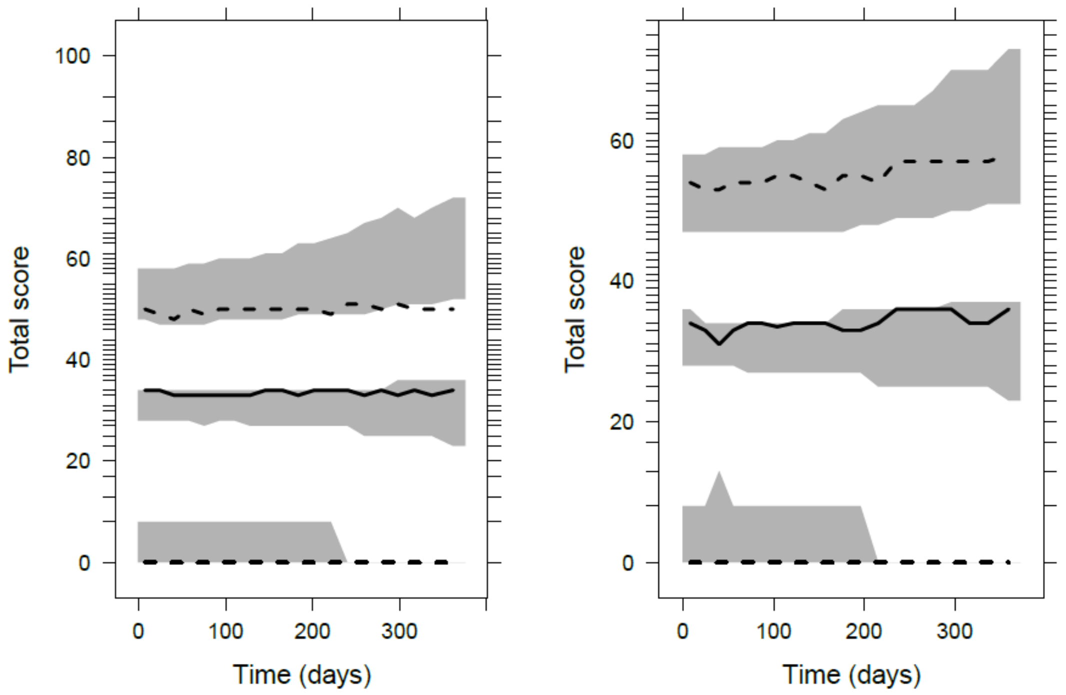

Figure S4: Visual predictive check (n=1000 simulations) for the total score (i.e. sum of item sub-scores of all items) stratified per sex: male (left), and female (right). Black lines represent data (2.5th, 50th, 97.5th percentiles), and grey areas are the corresponding 95% confidence intervals from 1,000 simulations.

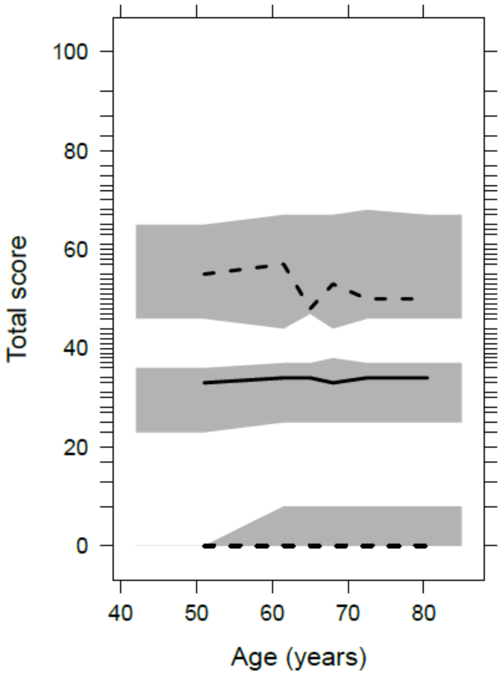

Figure S5: Visual predictive check (n=1000 simulations) for the total score (i.e. sum of item sub-scores of all items) versus age in years. Black lines represent data (2.5th, 50th, 97.5th percentiles), and grey areas are the corresponding 95% confidence intervals from 1,000 simulations.

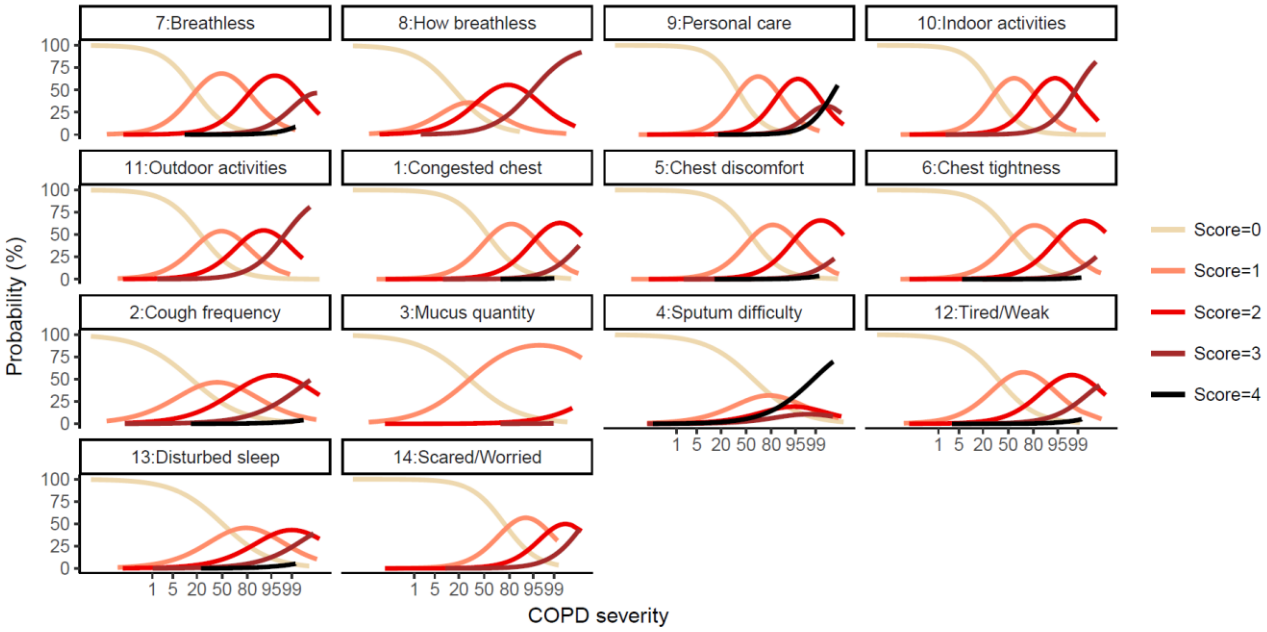

Figure S6: Item characteristic curves for all 14 items, plotted against chronic obstructive pulmonary disease (COPD) severity, represented in percentiles of the data (e.g. 50 indicates a typical subject from this population, lower numbers are 'healthier' part, and higher numbers are the 'sicker' part of the population). More detailed item descriptions are given in Table 1.
